# A *KCNJ10* mutation previously identified in the Russell group of terriers also occurs in Smooth-Haired Fox Terriers with hereditary ataxia and in related breeds

**DOI:** 10.1186/s13028-015-0115-1

**Published:** 2015-05-23

**Authors:** Cecilia Rohdin, Douglas Gilliam, Caroline A. O’Leary, Dennis P. O’Brien, Joan R. Coates, Gary S. Johnson, Karin Hultin Jäderlund

**Affiliations:** Department of Clinical Sciences, Swedish University of Agricultural Sciences, SE-750 07 Uppsala, Sweden; Anicura, Albano Small Animal Hospital, Rinkebyvägen 21, SE-182 36 Danderyd, Sweden; Department of Veterinary Pathobiology, University of Missouri, Columbia, MO USA; School of Veterinary Science, The University of Queensland, Gatton, QLD 4343 Australia; Department of Veterinary Medicine and Surgery, University of Missouri, Columbia, MO USA; Department of Companion Animal Clinical Sciences, Norwegian University of Life Sciences, P.O. Box 8146 Dep., NO-0033 Oslo, Norway

**Keywords:** Dog, Spinocerebellar ataxia, Myokymia, Smooth-Haired Fox Terrier, Toy Fox Terrier, Jack Russell Terrier, Parson Russell Terrier, Russell Terrier, Tenterfield Terrier, *KCNJ10*

## Abstract

**Background:**

Hereditary ataxias with similar phenotypes were reported in the Smooth-Haired Fox Terrier, the Jack Russell Terrier and the Parson Russell Terrier. However, segregation analyses showed differing inheritance modes in these breeds. Recently, molecular genetic studies on the Russell group of terriers found independent mutations in *KCNJ10* and *CAPN1*, each associated with a specific clinical subtype of inherited ataxia. The aim of this study was to clarify whether or not Smooth-Haired Fox Terriers with hereditary ataxia and dogs of other related breeds harbor either of the same mutations. A sub goal was to update the results of *KCNJ10* genotyping in Russell group terriers.

**Findings:**

Three Smooth-Haired Fox Terriers with hereditary ataxia and two Toy Fox Terriers with a similar phenotype were all homozygous for the *KCNJ10* mutation. The same mutation was also found in a heterozygous state in clinically unaffected Tenterfield Terriers (n = 5) and, in agreement with previous studies, in Jack Russell Terriers, Parson Russell Terriers, and Russell Terriers.

**Conclusions:**

A *KCNJ10* mutation, previously associated with an autosomal recessive spinocerebellar ataxia in Jack Russell Terriers, Parson Russell Terriers, and Russell Terriers segregates in at least three more breeds descended from British hunting terriers. Ataxic members of two of these breeds, the Smooth-Haired Fox Terrier and the Toy Fox Terrier, were homozygous for the mutation, strengthening the likelihood that this genetic defect is indeed the causative mutation for the disease known as “hereditary ataxia” in Fox Terriers and “spinocerebellar ataxia with myokymia, seizures or both” in the Russell group of terriers.

## Findings

The first report of a breed-related hereditary ataxia in the Smooth-Haired Fox Terrier was published in 1957 [1] and was followed in 1973 by the description of a disease with a similar phenotype in the Jack Russell Terrier [2]. In both breeds, the neurological signs were characterized by a prominent hypermetria along with a dancing and bouncing gait, with the onset of signs at 2- to 6-months of age. As these breeds have common ancestry, affected dogs were suspected to have the same disease. For both breeds, details of age of onset, clinical signs, histopathological changes and mode of inheritance were reported over the last decade [3–8]. However, segregation analyses indicated differing modes of inheritance in the Smooth-Haired Fox Terrier breed compared to the Jack Russell Terrier breed despite the phenotypic similarities between the diseases [1, 3]. In parallel, cases with similar neurologic phenotypes were also identified in other closely related breeds [unpublished observations].

Subsequently, Forman *et al*. [9] reported a missense mutation in the *CAPN1* gene that was strongly associated with a phenotypically similar inherited ataxia in the Parson Russell Terrier dog breed, with cases presenting at 6–12 months of age. However, some of the ataxic Parson Russell Terriers and Jack Russell Terriers included in the study were homozygous wild type for this mutation, indicating that more than one inherited ataxia might be segregating in the Russell group of terriers.

More recently a missense mutation in the *KCNJ10* gene [GenBank: XM_545752.3] was found to be significantly associated with a similar neurological disease in 14 ataxic dogs belonging to the Russell group of terriers [10]. All affected dogs exhibited a marked spinocerebellar ataxia with an onset of clinical signs between 2 and 6 months of age. Furthermore, myokymia, neuromyotonia, excessive facial rubbing, and seizures were observed in some cases. Affected dogs were homozygous for the mutation, strongly suggesting the genetic defect was causative for this autosomal recessive disorder. The authors proposed that this genetically defined disease be called “spinocerebellar ataxia with myokymia, seizures or both (SAMS)”, to distinguish this disorder from the late onset ataxia (LOA) reported by Forman *et al.* [9].

We began the present study by genotyping 6 neurologically affected dogs including three ataxic Smooth-Haired Fox Terriers, two ataxic Toy Fox Terriers, and a Danish-Swedish Farm Dog with neuromyotonia for the mutations associated with SAMS (*KCNJ10:c.627C>G*) and LOA (*CAPN1:c.344G>A*) to investigate whether the mutant alleles are associated with neurologic disease in these breeds that are related to the Russell group terriers. In each case, the DNA was extracted from EDTA-anti-coagulated blood. The three affected Smooth-Haired Fox Terriers had been diagnosed with hereditary ataxia. Two of them were Swedish dogs, reported in Rohdin *et al*. [5] as A1 and A2. The third Smooth-Haired Fox Terrier was a male from Norway that presented at 11-months of age with myokymia and neuromyotonic attacks and had a 7–8 month history of pronounced hypermetria. These three Smooth-Haired Fox Terriers were all related through both their fathers’ and their mothers’ lineages to a specific ancestor, identified by Björck [personal communication] as a carrier of the familial ataxia that he had investigated [1, 11]. In addition, in 2014 we obtained blood samples from two American Toy Fox Terriers, both with SAMS-like clinical signs. One was a rescue dog that was recognized as a Toy Fox Terrier because of his size and appearance. The other Toy Fox Terrier was bred and owned by an established Toy Fox Terrier breeder and was registered as a Toy Fox Terrier with the American Kennel Club. We also obtained a blood sample from a Danish-Swedish Farm Dog with a complex history. The dog was diagnosed at a young age with aortic stenosis and a tracheal malformation, had “always” been clumsy, and had shown muscle twitches at rest. At 4 years of age, the dog was presented for a neurological examination and displayed neuromyotonia, myokymia and excessive facial rubbing. The dog was not ataxic at this examination. In addition, we have included *KCNJ10:c.627C>G* and *CAPN1:c.344G>A* genotype results for all 22 of the Russell group terriers with SAMS-like signs that are represented in our DNA collection. This cohort consists of 18 ataxic Jack Russell Terriers and 4 ataxic Parson Russell Terriers and is an update from a previously communication [10] that reported the genotypes of 16 of these ataxic Russell-group terriers.

Twenty four of the 28 dogs with neurologic disease were homozygous for the mutant *KCNJ10:c.627G* allele. This included all three ataxic Smooth-Haired Fox Terriers, both ataxic Toy Fox Terriers, all 4 of the ataxic Parson Russell Terriers, and 15 of the 18 ataxic Jack Russell Terriers. The occurrence of SAMS-like signs in all 24 of the *KCNJ10:c.627G* homozygotes; indicates that the disease caused by *KCNJ10:c.627G* homozygosity is highly penetrant.

All of the 28 dogs with neurologic disease were homozygous for the wild-type *CAPN1:c.344G* allele. Thus, three of the ataxic Jack Russell Terriers and the Danish-Swedish-Farm Dog with neuromyotonia, myokymia and facial rubbing were homozygous for the wild-type alleles at both *CAPN1:c.344G>A* and *KCNJ10:c.627C>G*. The identification of phenocopies in these breeds suggests the existence of additional inherited or acquired causes for SAMS-like clinical signs.

It is likely that the *KCNJ10:c.627G*-homozygous Fox terriers share ancestry with the *KCNJ10:c.627G*-homozygous Russell group terriers because both the Fox terriers and the Russell group terriers are believed to have descended from the British hunting terrier. We expanded the study to see if we could find the *KCNJ10:c.627G* allele in other related breeds. Specifically, we generated *KCNJ10:c.627C>G* genotypes for members of 19 other breeds with likely British hunting terrier ancestry. Initially, the selection of samples for genotyping was based only on breed, without regard to disease phenotype. Later, the available clinical records were reviewed. If the records indicated that the dogs were reported by their owners or their veterinarians to be neurologically normal at or after one year of age, the dogs were classified as “ataxia-free.” None of the clinical records indicated that the selected samples were from dogs that exhibited SAMS-like signs; however, clinical records for many of dogs with selected samples were absent or uninformative. These dogs were classified as “phenotype unknown.”

Finally, we have included all Russell group terrier *KCNJ10:c.627C>G* genotypes determined at the University of Missouri before the end of November, 2014. These include genotypes for the earlier mentioned 18 ataxic Jack Russell Terriers and the 4 ataxic Parson Russell Terriers, as well as for the 1786 Jack Russell, Parson Russell and Russell Terriers without a known history of ataxia. The clinical records for these dogs were reviewed and the dogs were classified as “SAMS-like signs,” “ataxia free,” or “phenotype unknown.”

The distribution of genotypes among each phenotype classification is presented in Table 1. Note that none of the *c.627C/G* heterozygotes had SAMS-like signs. This supports our earlier assertion that the *KCNJ10:c.627G*-associated disease phenotype is a recessive trait [10]. Table 2 shows the distribution of *KCNJ10:c.627C>G* genotypes among the various dog breeds. As discussed earlier, clinically affected *KCNJ10:c.627G* homozygotes were identified in four breeds: the Jack Russell Terrier, the Parson Russell Terrier, the Smooth-Haired Fox Terrier, and the Toy Fox Terrier. *KCNJ10:c.627C/G* heterozygotes were identified among clinically normal members of two additional breeds: the Russell Terrier and the Tenterfield Terrier. The G-allele frequencies for the tested cohorts from these two breeds are similar to the G-allele frequencies of the tested cohorts of the four breeds with already identified clinically affected G-allele homozygotes. Thus, it is likely that *c.627G* homozygous Russell Terriers and Tenterfield Terriers exist but have not yet been reported. All 128 representatives of 19 other breeds believed to have British hunting dog ancestry were homozygous for the wild type *c.627C* allele; however, for many of these breeds, very few dogs were genotyped. Thus, it is plausible that additional testing could identify carriers of the *c.627G* allele in some of these breeds and in other related breeds.

**Table 1:** Distribution *KCNJ10:c.627C>G* genotypes among the various SAMS-related clinical phenotypes

		Clinical phenotype			
		SAMS-like signs	Ataxia free	Phenotype unknown	Total
Genotype	Wt^a^/Wt	4	842	957	1803
	Mut^a^/Wt	0	86	139	225
	Mut/Mut	24	0	0	24
	Total	28	928	1096	2052

^a^Wt = wild type allele, Mut = mutant allele

**Table 2:** The distribution of 2052 *KCNJ10:c.627C>G* genotypes in 25 different dog breeds

Breed	Mut^a^/Mut	Mut/Wt^a^	Wt/Wt	Number tested	G allele frequency	Home country of dogs carrying the Mut allele (Mut/Mut, Mut/Wt)
Jack Russell Terrier	15	153	1071	1239	0.074	USA (13,144), Canada (0,3), France (1,0), Germany (1,6)
Parson Russell Terrier	4	51	429	484	0.061	USA (3,36), Canada (0,1), Germany (1,13), Norway (0,1)
Russell Terrier	0	12	73	85	0.071	USA (0,11), Canada (0,1)
Smooth-Haired Fox Terrier	3	0	33	36	0.083	Sweden (2,0), Norway (1,0)
Tenterfield Terrier	0	5	52	57	0.044	Australia (0,5)
Toy Fox Terrier	2	4	17	23	0.174	USA (2,4)
Other Breeds^b^	0	0	128	128	0	
Total	24	225	1803	2052		

^a^Mut = mutated allele, Wt = wild type allele

^b^Breed (number tested): Airedale Terrier (4), American Hairless Terrier (3), American Staffordshire Terrier (2), Australian Cattle Dog (3), Border Terrier (12), Bull Terrier (1), Danish-Swedish Farm Dog (1), Decker Terrier (1), Irish Terrier (1), Jagdterrier (31), Kerry Blue Terrier (3), Manchester Terrier (2), Miniature Bull Terrier (48), Pit Bull Terrier (3), Rat Terrier (1), Silky Terrier (1), Soft Coated Wheaten Terrier (5), Staffordshire Bull Terrier (1), Wirehaired Fox Terrier (5)

In conclusion, the *KCNJ10* mutation previously associated with SAMS in the Russell group of terriers was also found in the ataxic Smooth-Haired Fox Terriers and Toy Fox Terriers examined in this study. Identification of this mutation in a homozygous state in ataxic dogs of different, albeit related, breeds supports an earlier assertion [10] that this mutation is causal for a neurodegenerative disease that is inherited in an autosomal recessive mode. In addition, the mutant allele has been identified in a heterozygous state in clinically unaffected members of other breeds believed to have similar ancestry, suggesting that this disease could also be present in these related breeds. Thus, a *KCNJ10* mutation should be considered as a potential cause for ataxias with onsets between 2 and 6 months of age, particularly if they occur in members of breeds with British hunting terrier ancestry and if the ataxias are accompanied by one or more of the following: myokymia, neuromyotonia, seizures, and face rubbing.

## Abbreviations

SAMS: Spinocerebellar ataxia with myokymia, seizures, or both
LOA: Late onset ataxia
